# Molecular characterization of multidrug-resistant non-typeable *Haemophilus influenzae* with high-level resistance to cefuroxime, levofloxacin, and trimethoprim-sulfamethoxazole

**DOI:** 10.1186/s12866-023-02926-6

**Published:** 2023-07-05

**Authors:** Pei-Yi Su, Wei-Hung Cheng, Cheng-Hsun Ho

**Affiliations:** 1 Department of Laboratory Medicine, E-Da Hospital, Kaohsiung, Taiwan; 2 Department of Parasitology, College of Medicine, National Cheng Kung University, Tainan, Taiwan; 3 Department of Medical Laboratory Science, College of Medical Science and Technology, I-Shou University, No.8, Yida Road, Jiaosu Village, Yanchao District, Kaohsiung City, 82445 Taiwan

**Keywords:** Non-typeable *Haemophilus influenzae*, Antimicrobial susceptibility test, Multidrug-resistant, Whole-genome analysis

## Abstract

**Background:**

Non-typeable *Haemophilus influenzae* (NTHi) has become the major cause of invasive *H. influenzae* diseases in the post-*H. influenzae* type b vaccine era. The emergence of multidrug-resistant (MDR) NTHi is a growing public health problem. Herein, we investigated the molecular basis of MDR in NTHi. The isolated NTHi were subjected to antimicrobial susceptibility testing for 12 agents. Whole genome and plasmid sequencing were conducted and analyzed to identify significant genetic variations and plasmid-encoded genes conferred antibiotic resistance.

**Results:**

Thirteen (50%) MDR NTHi isolates were obtained; of these, 92.3% were non-susceptible to ampicillin, 30.8% to amoxicillin-clavulanate, 61.5% to cefuroxime, 61.5% to ciprofloxacin/levofloxacin, 92.3% to trimethoprim-sulfamethoxazole, 30.8% to tetracycline, and 7.7% to azithromycin. Eight ampicillin-resistant isolates were β-lactamase positive; of these, 6 carried *bla*_TEM-1_ and 2 carried *bla*_ROB-1_, whereas 4 were β-lactamase negative. Genetic variations in *mrdA*, *mepA*, and *pbpG* were correlated with amoxicillin-clavulanate non-susceptibility, whereas variations in *ftsI* and *lpoA* conferred cefuroxime resistance. Five variations in *gyrA*, 2 in *gyrB*, 3 in *parC*, 1 in *parE*, and 1 in the *parC*-*parE* intergenic region were associated with levofloxacin/ciprofloxacin non-susceptibility. Among these genes, 8 variations were linked to high-level levofloxacin resistance. Six variations in *folA* were associated with trimethoprim-sulfamethoxazole resistance. Plasmid-bearing *tet(B)* and *mef(A)* genes were responsible for tetracycline and azithromycin resistance in 4 and 1 MDR isolates, respectively.

**Conclusions:**

This study clarified the molecular epidemiology of MDR in NTHi. This can benefit the monitoring of drug resistance trends in NTHi and the adequate medical management of patients with NTHi infection.

**Supplementary Information:**

The online version contains supplementary material available at 10.1186/s12866-023-02926-6.

## Introduction

The global vaccination programme against *Haemophilus influenzae* type b (Hib) has substantially affected the epidemiology of Hib infections in most countries. Compelling observations provide concrete evidence for the thriving of non-typeable *H. influenzae* (NTHi) in the post-Hib vaccination era. NTHi is a major cause of otitis media, bacterial sinusitis, and bacterial conjunctivitis in children; moreover, it exacerbates chronic obstructive pulmonary disease and causes up to 5% of neonatal invasive bacterial infections [1]. National surveillance data from 14 European and other countries indicate that NTHi causes 97% of non-Hib infections in invasive *H. influenzae* disease [2]. The highest incidence of invasive NTHi diseases occurs in premature infants, elderly adults, the immunosuppressed, individuals with malignant disease, and those with chronic cardiovascular or respiratory conditions. The fatality rate is higher than 10% among such individuals [3, 4].

Empirical treatment for NTHi infection is akin to that for typeable *H. influenzae* infection. Ampicillin is the standard recipe for β-lactamase-negative ampicillin-susceptible NTHi infections, with chloramphenicol or broad-spectrum cephalosporins used as alternate regimens. In both typeable *H. influenzae* and NTHi, β-lactamase production, particularly by *bla*_TEM-1_, is the predominant resistance mechanism against penicillins and some cephalosporins. Studies have reported rates of β-lactamase-positive ampicillin-resistant (BLPAR) NTHi between 10 and 25% in South Africa, Europe, and the Americas [5–8]. In Taiwan, Vietnam, Japan, and South Korea, BLPAR may constitute up to 55% of NTHi [9–12]. The prevalence of β-lactamase-negative ampicillin-resistant (BLNAR) NTHi is also high in Taiwan, Vietnam, and South Korea [9, 11, 12]. Macrolides, quinolones, tetracycline, trimethoprim-sulfamethoxazole, and third-generation cephalosporins may provide coverage for ampicillin-resistant *H. influenzae* infection.

Multidrug-resistant (MDR) NTHi was infrequent in Western countries but has been sporadically reported in Asian areas [13–15]. Reported MDR NTHi strains were resistant to β-lactams, macrolides, quinolones, tetracycline, and trimethoprim-sulfamethoxazole, suggesting that NTHi is acquiring drug resistance through different ploys. Because the molecular epidemiology of MDR in NTHi remains unclear, we conducted whole genome and plasmid analyses to delineate the genes and genetic variations relevant to MDR NTHi and high-level drug resistance.

## Results

### Antibiogram of NTHi

**Fig. 1:**
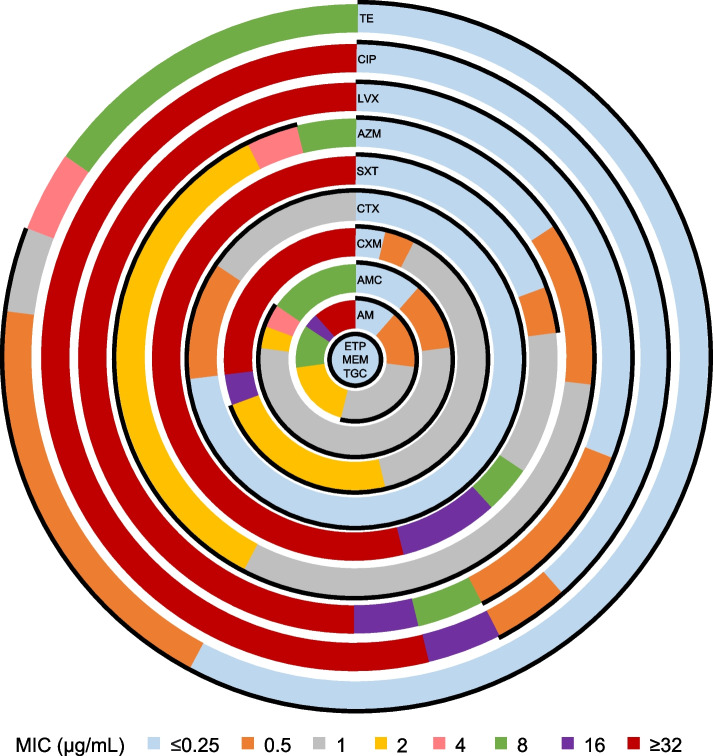
MIC and susceptibility profile of non-typeable *Haemophilus influenzae* (NTHi). Percentage of MICs of different antimicrobial agents in 26 NTHi isolates are shown in a circle plot. Bold arc represents the drug-susceptible range according to the Clinical & Laboratory Standards Institute M100 31st edition. MICs of ertapenem, meropenem, and tigecycline in all isolates were ≤ 0.25 μg/mL. AM, ampicillin; AMC, amoxicillin-clavulanate; AZM, azithromycin; CIP, ciprofloxacin; CTX, cefotaxime; CXM, cefuroxime; ETP, ertapenem; LVX, levofloxacin; MEM, meropenem; MIC, minimum inhibitory concentration; SXT, trimethoprim-sulfamethoxazole; TE, tetracycline; TGC, tigecycline

Twenty-six NTHi isolates were included in this study. An analysis of β-lactam resistance revealed that 12 (46.2%) isolates were non-susceptible to ampicillin, 4 (15.4%) to amoxicillin-clavulanate, and 8 (30.8%) to cefuroxime (Fig. 1). For non-β-lactam resistance, 20 (76.9%) isolates were non-susceptible to trimethoprim-sulfamethoxazole, 1 (3.8%) to azithromycin, 15 (57.7%) to levofloxacin and ciprofloxacin, and 5 (19.2%) to tetracycline. All isolates were susceptible to cefotaxime, ertapenem, meropenem, and tigecycline. Regarding the antibiogram, 5 isolates (No. 01, 11, 12, 14, and 20) were susceptible to all drugs, whereas 8 were non-susceptible to 2 categories of agents (Table 1). Among 13 MDR isolates, 4 (No. 05, 08, 13, and 18) were non-susceptible to 3 categories of agents, 8 (No. 04, 06, 07, 09, 10, 15, 19, and 23) to 4 categories, and 1 (No. 22) to 5 categories.

**Table 1:** Antibiogram of non-typeable *Haemophilus influenzae* isolates

	Categories, non-susceptible									
Isolate	Penicillins	β-lactam combination agents	Cephems	Carbapenems	Fluoroquinolones	Folate pathway antagonists	Macrolides	Tetracyclines	Glycylcyclines	MDR
ED01										
ED02						SXT		TE		
ED03					CIP, LVX	SXT				
ED04	AM				CIP, LVX	SXT	AZM			V
ED05	AM				CIP, LVX	SXT				V
ED06	AM	AMC	CXM			SXT				V
ED07	AM		CXM			SXT		TE		V
ED08	AM					SXT		TE		V
ED09	AM		CXM		CIP, LVX	SXT				V
ED10	AM				CIP, LVX	SXT		TE		V
ED11										
ED12										
ED13	AM	AMC	CXM							V
ED14										
ED15	AM				CIP, LVX	SXT		TE		V
ED16					CIP, LVX	SXT				
ED17					CIP, LVX	SXT				
ED18			CXM		CIP, LVX	SXT				V
ED19	AM	AMC	CXM			SXT				V
ED20										
ED21					CIP, LVX	SXT				
ED22	AM	AMC	CXM		CIP, LVX	SXT				V
ED23	AM		CXM		CIP, LVX	SXT				V
ED24					CIP, LVX	SXT				
ED25					CIP, LVX	SXT				
ED26					CIP, LVX	SXT				

*Abbreviations: AM* Ampicillin, *AMC* Amoxicillin-clavulanate, *AZM* Azithromycin, *CIP* Ciprofloxacin, *CXM* Cefuroxime, *LVX* Levofloxacin, *MDR* Multidrug-resistant, *SXT* Trimethoprim-sulfamethoxazole, *TE* Tetracycline

### Plasmid-mediated drug resistance

Plasmid-encoded antibiotic resistance genes were assessed in each isolate. An intact *bla*_TEM-1_ was detected in 6 isolates (Table 2). Partial sequences of *bla*_TEM-1_ were detected in isolate No. 23. Moreover, genes with high sequence identity to *bla*_ROB-1_ were discovered in isolates No. 22 and 23; *bla*_ROB-11_ in isolate No. 22. Two of these BLPAR isolates (No. 06 and 22) were β-lactamase-positive amoxicillin-clavulanate resistant. Among 5 tetracycline-resistant isolates, *tetR*/*acrR* family transcriptional regulator gene was detected in isolate No. 02 and *tet(B)* in the other 4 isolates. Moreover, *mef(A)* was discovered in the azithromycin-non-susceptible isolate (No. 04). No plasmid-mediated quinolone-resistance genes or trimethoprim-sulfamethoxazole-resistance genes were characterized.

**Table 2:** Identification of plasmid-encoded drug-resistant gene in non-typeable *Haemophilus influenzae* isolates

Isolate	Identification	Accession	Greatest identity (%)	Lowest E-value
*β-lactamase*				
ED04	TEM-1	WP_000027057.1	100.0	0
ED06	TEM-1	WP_000027057.1	100.0	0
ED07	TEM-1	WP_000027057.1	100.0	0
ED08	TEM-1	WP_000027057.1	100.0	0
ED10	TEM-1	WP_000027057.1	100.0	0
ED15	TEM-1	WP_000027057.1	100.0	0
ED22	ROB-1	WP_005618523.1	90.7	5.2E-64
	ROB-11	WP_132995818.1	90.5	7.0E-40
ED23	ROB-1	WP_005618523.1	99.3	0
	TEM-1 (aa 150–185)	WP_000027057.1	91.7	9.0E-22
	TEM-1 (aa 274–286)	WP_000027057.1	84.6	2.0E-06
*Tetracycline efflux*				
ED02	TetR/AcrR family	WP_005751845.1	59.1	2.2E-53
ED07	Tet(B)	WP_001089072.1	99.8	0
ED08	Tet(B)	WP_001089072.1	99.8	0
ED10	Tet(B)	WP_001089068.1	100.0	0
ED15	Tet(B)	WP_001089072.1	100.0	0
*Macrolide efflux*				
ED04	Mef(A)	WP_024410015.1	100.0	6.0E-41

Drug-resistant genes were assessed using BLASTx and compared with sequences available in the RefSeq database of the National Center for Biotechnology Information

*Abbreviation: aa* Amino acid

### Genomic variations associated with drug resistance

In the analysis of β-lactam resistance, we focused on penicillin-binding proteins (PBPs) and penicillin-insensitive-related proteins. In ampicillin-resistant isolates, the group I *ftsI* mutation was detected in 2 BLPAR isolates, and both group II and III *ftsI* mutations were detected in 3 BLPAR isolates (Table 3). In BLNAR isolates, group II *ftsI* mutation was detected in isolate No. 05, and group III-like *ftsI* mutation was observed in isolates No. 09 and 13. A novel *ftsI* mutation type was found in isolate No. 19. Furthermore, genetic variations, namely 1070G > A (Ser357Asn), 1594A > T (Thr532Ser), and 1669T > C (Tyr557His) in *ftsI*, and 453G > C (Met151Ile) in *lpoA* (PBP1a), were associated with cefuroxime-resistance (Table 4). The area under the ROCs (AUROCs) for Ser357Asn, Thr532Ser, and Tyr557His substitutions in PBP3 and Met151Ile in PBP1a to discriminate the MIC of cefuroxime were 0.805, 0.910, 0.946, and 0.891, respectively. Other than *ftsI*, 3 genetic variations, namely 1552G > A in *HI_0032 (mrdA)* for Ala518Thr substitution in PBP2, 82C > A in *HI_0197 (mepA)* for Gln28Lys in penicillin-insensitive murein endopeptidase, and 674G > T in *HI_0364 (pbpG)* for Arg225Leu in PBP7, were correlated with amoxicillin-clavulanate non-susceptibility in NTHi (Table 4). The AUROCs for Ala518Thr substitution in PBP2, Gln28Lys in penicillin-insensitive murein endopeptidase, and Arg225Leu in PBP7 to discriminate the MIC of amoxicillin-clavulanate were 0.750, 0.767, and 0.859, respectively.

**Table 3:** Amino acid substitutions in FtsI in ampicillin-resistant non-typeable *Haemophilus influenzae* isolates

	D	S	M	S	L	G	A	R	N	T	V	Y	V	N	A	N	T	S	A	I	E	
Isolate	350	357	377	385	389	490	502	517	526	532	547	557	562	569	586	589	591	594	595	601	603	Group
*BLPAR*																						
ED04	N		I				V		K		I			S	S			T	T			II
ED06^ab^	N	N	I	T	F				K		I		L	S								III
ED07^b^	N	N	I	T	F		V		K		I			S	S			T	T		D	III
ED08	N		I			E	V		K		I			S		K	A			V	D	II
ED10	N	N	I	T	F				K		I		L	S	S			T	T	V	D	III
ED15	N						V		K		I	D		S	S			T	T			II
ED22^ab^	N	N	I	N	F			H		S	I	H		S	P			T	T		D	I
ED23^b^	H							H		A	I	H		S							K	I
*BLNAR*																						
ED05	N						V		K		I			S	S			T	T			II
ED09^b^	N	N	I	T	F			H		S	I	H		S	S			T	T			III-like
ED13^ab^	N	N	I	T	F			H		S	I	H		S	S			T	T		D	III-like
ED19^ab^	N	N		I					H		L		L			K						ND

Group I, R517H; group II, N526K; group III, N526K with S385T; group III-like, R517H with S385T

^a^Amoxicillin-clavulanate-non-susceptible; ^b^Cefuroxime-resistant

*Abbreviations: BLNAR* β-lactamase-negative ampicillin-resistance, *BLPAR* β-lactamase-positive ampicillin-resistance, *ND* Not determined

**Table 4:** Genetic variations associated with cefuroxime, amoxicillin-clavulanate, ciprofloxacin/levofloxacin, and trimethoprim-sulfamethoxazole non-susceptibility in non-typeable *Haemophilus influenzae* isolates

Variant (isolate detected, n)	Fisher’s exact test			ROC curve			
	Susceptible	Non-susceptible	*P*-value	AUROC (SE)	Sensitivity	Specificity	*P*-value
Cefuroxime	18	8					
* HI_1132 (ftsI)*							
1070G > A (Ser357Asn)	6	7	0.030	0.805 (0.087)	76.9%	69.2%	0.008
1594A > T (Thr532Ser)	1	4	0.020	0.910 (0.066)	80.0%	85.7%	0.005
1669 T > C (Tyr557His)	1	5	0.004	0.946 (0.050)	83.3%	90.0%	0.001
* HI_1655 (lpoA)*							
453G > C (Met151Ile)	0	3	0.022	0.891 (0.071)	100.0%	82.6%	0.030
Amoxicillin-clavulanate	22	4					
* HI_0032 (mrdA)*							
1552G > A (Ala518Thr)	6	4	0.014	0.750 (0.101)	50.0%	100.0%	0.035
* HI_0197 (mepA)*							
82C > A (Gln28Lys)	4	3	0.047	0.767 (0.125)	57.1%	94.7%	0.040
* HI_0364 (pbpG)*							
674G > T (Arg225Leu)	6	4	0.014	0.859 (0.073)	70.0%	81.2%	0.002
Ciprofloxacin / Levofloxacin	11	15					
* HI_1264 (gyrA)*							
251C > T (Ser84Phe)	4	13	0.014	0.752 (0.103)	86.7%	63.6%	0.031
263A > G (Asp88Gly)	0	7	0.010	0.733 (0.098)	46.7%	100.0%	0.046
1299G > T (Glu433Asp)	1	9	0.014	0.755 (0.097)	60.0%	90.9%	0.029
1414A > G (Ile472Val)	1	10	0.005	0.788 (0.092)	66.7%	90.9%	0.014
2220 T > A (Asp740Glu)	1	8	0.036	0.721 (0.101)	53.3%	90.9%	0.058
* HI_0567 (gyrB)*							
1817C > T (Ala606Val)	5	13	0.038	0.706 (0.109)	86.7%	54.5%	0.078
2174C > T (Ala725Val)	1	9	0.014	0.755 (0.097)	60.0%	90.9%	0.029
* HI_1529 (parC)*							
59A > G (Lys20Arg)	0	11	< 0.001	0.867 (0.074)	73.3%	100.0%	0.002
251G > T (Ser84Ile)	1	11	0.002	0.821 (0.087)	73.3%	90.9%	0.006
1601G > A (Ser534Asn)	0	6	0.024	0.700 (0.102)	40.0%	100.0%	0.087
* HI_1528 (parE)*							
434G > A (Ser145Asn)	0	8	0.007	0.767 (0.094)	53.3%	100.0%	0.022
* parC*-*parE* intergenic region							
n.1600250A > C	0	11	< 0.001	0.867 (0.074)	73.3%	100.0%	0.002
Trimethoprim-sulfamethoxazole	6	20					
* HI_0899 (folA)*							
38A > G (Asn13Ser)	1	19	< 0.001	0.954 (0.047)	100.0%	83.3%	< 0.001
63G > A (Met21Ile)	0	11	0.024	0.585 (0.114)	100.0%	40.0%	0.467
200T > C (Leu67Pro)	0	13	0.015	0.654 (0.112)	100.0%	46.2%	0.182
250A > T (Asn84Tyr)	0	11	0.024	0.585 (0.114)	100.0%	40.0%	0.467
283A > C (Ile95Leu)	1	17	0.004	0.795 (0.112)	100.0%	62.5%	0.018
319A > C (Lys107Gln)	0	16	< 0.001	0.775 (0.106)	100.0%	60.0%	0.020

ROC analyses show the power of each genetic variant for differentiating the minimum inhibitory concentration

*Abbreviations*: *AUROC* Area under ROC curve, *ROC* Receiver operating characteristic, *SE* Standard error

In isolates non-susceptible to fluoroquinolones and folate pathway antagonists, 12 genetic variations in *gyrA*, *gyrB*, *parC*, *parE*, and the *parA*-*parC* intergenic region and 6 variations in *folA* were associated with ciprofloxacin/levofloxacin and trimethoprim-sulfamethoxazole non-susceptibility, respectively (Table 4). The AUROCs for Ser84Phe, Asp88Gly, Glu433Asp, and Ile472Val substitutions in GyrA, Ala725Val in GyrB, and Ser145Asn in ParE to discriminate the MIC of levofloxacin were all higher than 0.73. Corresponding AUROCs for Lys20Arg and Ser84Ile substitutions in ParC and n.1600250A > C in the *parC*-*parE* intergenic region were higher than 0.82. Furthermore, AUROCs for Asn13Ser, Ile95Leu, and Lys107Gln substitutions in FolA to discriminate the MIC of trimethoprim-sulfamethoxazole were 0.954, 0.795, and 0.775, respectively.

We analyzed the sequence of related transporter and porin genes in the NTHi isolates (Table S1). No genetic variations in these genes were associated with the resistance to the agents we tested.

### Protein substitutions associated with high-level drug resistance

**Fig. 2:**
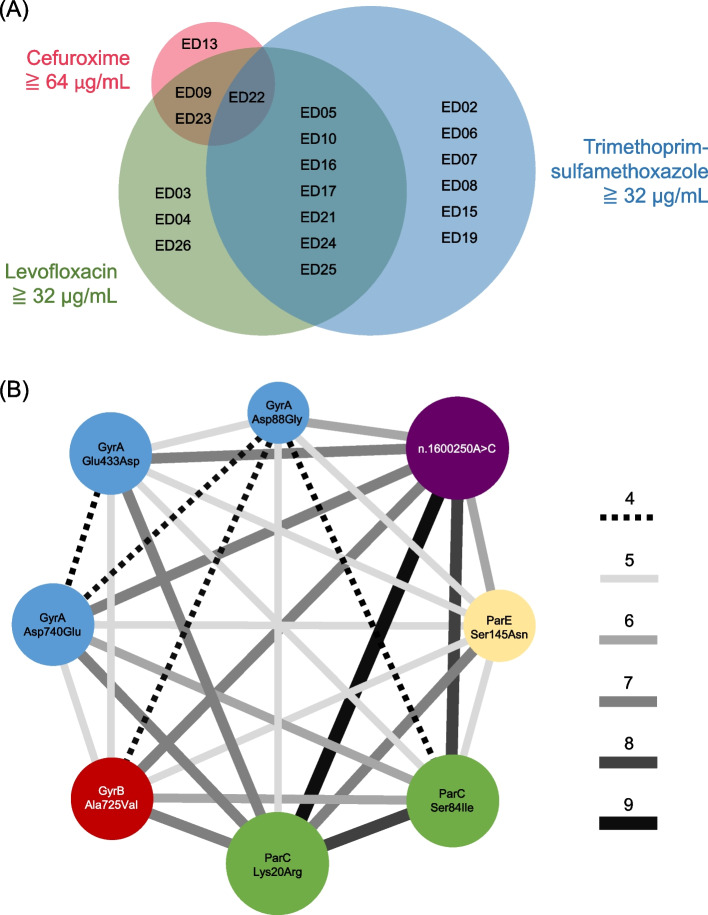
Isolates with high-level resistance to cefuroxime, levofloxacin, and trimethoprim-sulfamethoxazole. (A) A Venn diagram for NTHi isolates with cefuroxime MIC ≧64 μg/mL and both levofloxacin and trimethoprim-sulfamethoxazole MICs ≧32 μg/mL is shown. (B) A network clustering of mutation for high-level levofloxacin resistance is shown. The size of the circles and width of the lines are proportional to the frequency of each mutation and the connection of two mutations, respectively

Four NTHi isolates had a high-level cefuroxime resistance (MIC ≧64 μg/mL) (Fig. 2A). Moreover, 13 and 14 isolates had high-level resistance to levofloxacin (MICs ≧32 μg/mL) and trimethoprim-sulfamethoxazole (MICs ≧32 μg/mL), respectively. A logistic regression analysis revealed that Thr532Ser substitution in FtsI was an independent factor associated with high-level cefuroxime resistance (Table 5). Furthermore, Asp88Gly, Glu433Asp, and Asp740Glu substitutions in GyrA, Ala725Val in GyrB, Lys20Arg and Ser84Ile in ParC, Ser145Asn in ParE, and n.1600250A > C in the *parC*-*parE* intergenic region held up the relationship to high-level levofloxacin resistance. A network clustering showed the connection of these 8 variations (Fig. 2B). No single mutation was found to be associated with high-level trimethoprim-sulfamethoxazole resistance.

**Table 5:** Logistic regression analyses of genetic variations associated with high-level resistance to cefuroxime, levofloxacin, and trimethoprim-sulfamethoxazole in NTHi

Variable	Odds ratio (95% CI)	*P*-value
Cefuroxime		
*HI_1132 (ftsI)*		
1070G > A (Ser357Asn)	3.600 (0.322 – 40.233)	0.298
1594A > T (Thr532Ser)	30.000 (2.037 – 441.839)	0.013
1669 T > C (Tyr557His)	3.23E09 (0.000 – 0.000)	0.998
*HI_1655 (lpoA)*		
453G > C (Met151Ile)	3.55E10 (0.000 – 0.000)	0.999
Levofloxacin		
*HI_1264 (gyrA)*		
251C > T (Ser84Phe)	6.417 (0.999 – 41.212)	0.050
263A > G (Asp88Gly)	10.286 (1.018 – 103.948)	0.048
1299G > T (Glu433Asp)	8.800 (1.349 – 57.426)	0.023
1414A > G (Ile472Val)	5.333 (0.968 – 29.393)	0.055
2220 T > A (Asp740Glu)	19.200 (1.876 – 196.539)	0.013
*HI_0567 (gyrB)*		
1817C > T (Ala606Val)	4.714 (0.734 – 30.278)	0.102
2174C > T (Ala725Val)	8.800 (1.349 – 57.426)	0.023
*HI_1529 (parC)*		
59A > G (Lys20Arg)	40.000 (3.579 – 477.034)	0.003
251G > T (Ser84Ile)	7.500 (1.307 – 43.028)	0.024
1601G > A (Ser534Asn)	7.500 (0.733 – 76.773)	0.090
*HI_1528 (parE)*		
434G > A (Ser145Asn)	14.000 (1.385 – 141.485)	0.025
*parC*-*parE* intergenic region		
n.1600250A > C	40.000 (3.579 – 477.034)	0.003
Trimethoprim-sulfamethoxazole		
*HI_0899 (folA)*		
38A > G (Asn13Ser)	3.77E9 (0.000 – 0.000)	0.999
63G > A (Met21Ile)	1.364 (0.188 – 9.912)	0.759
200T > C (Leu67Pro)	1.867 (0.392 – 8.894)	0.433
250A > T (Asn84Tyr)	1.050 (0.220 – 5.003)	0.951
283A > C (Ile95Leu)	6.000 (0.919 – 39.185)	0.061
319A > C (Lys107Gln)	5.133 (0.922 – 28.570)	0.062

High-level resistance to cefuroxime, levofloxacin, and trimethoprim-sulfamethoxazole were defined as MIC of ≧64 μg/mL, ≧32 μg/mL, and ≧32 μg/mL, respectively

*Abbreviation*: *CI* Confidence interval, *NTHi* Non-typeable *Haemophilus influenzae*

### Molecular epidemiology of MDR NTHi

**Fig. 3:**
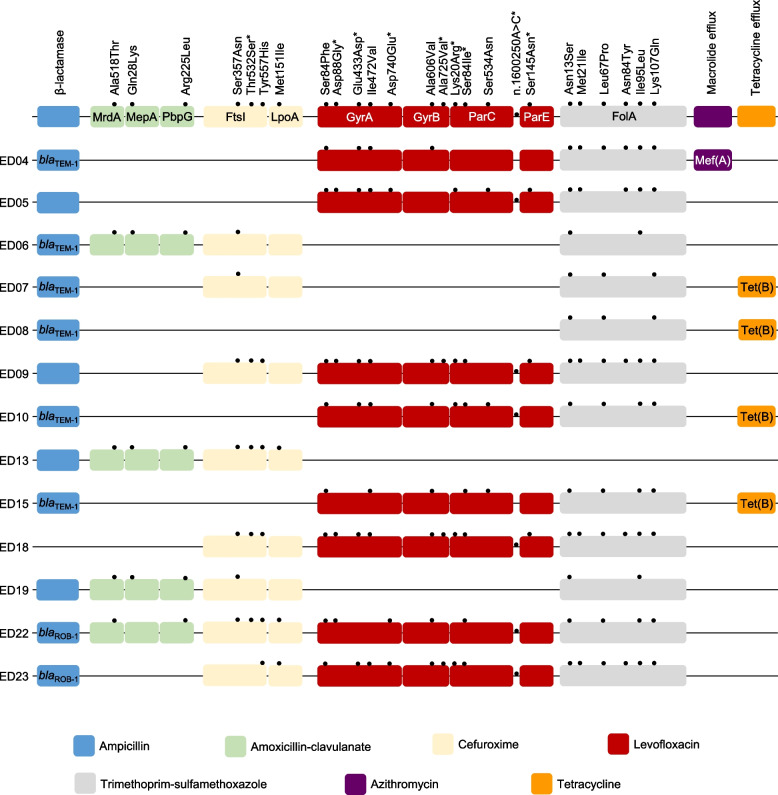
Schematic diagram of amino acid substitution panels in multidrug-resistant non-typeable *Haemophilus influenzae* isolates. Blue box, ampicillin-resistance; green box, amoxicillin-clavulanate resistance; beige box, cefuroxime resistance; red box, levofloxacin resistance; gray box, trimethoprim-sulfamethoxazole resistance; purple box, azithromycin resistance; orange box, tetracycline resistance. Substitutions associated with high-level drug resistance were marked with an asterisk

The molecular epidemiology of antimicrobial resistance in 13 MDR NTHi isolates is displayed in Fig. 3. Ampicillin resistance was detected in 12 isolates; of these 8 were BLPAR (*bla*_TEM-1_ in 6 and *bla*_ROB-1_ in 2), and 4 were BLNAR. Amino acid substitutions in FtsI in ampicillin-resistant isolates are listed in Table 3. Thr532Ser, Ser357Asn, and Tyr557His substitutions in FtsI were detected in 7, 4, and 5 cefuroxime-resistant MDR isolates, respectively. Furthermore, Ala518Thr substitution in MrdA and Arg225Leu in PbpG were detected in all amoxicillin-clavulanate-non-susceptible MDR isolates, whereas Gln28Lys in MepA was absent in isolate No. 22. Regarding levofloxacin and ciprofloxacin non-susceptibility, Ser84Phe, Asp88Gly, Glu433Asp, Ile472Val, and Asp740Glu substitutions in GyrA were observed in 8, 4, 6, 7, and 3 isolates, respectively. Moreover, Ala606Val substitution in GyrB, Ser84Ile on ParC, and n.1600250A > C in the *parC*-*parE* intergenic region were discovered in more than 75% of MDR isolates. Asn13Ser substitution in FolA was found in all 12 trimethoprim-sulfamethoxazole-resistant MDR isolates, and Leu67Pro, Ile95Leu, and Lys107Gln in more than 9 MDR isolates. Finally, Tet(B), which confers tetracycline resistance was detected in 4 MDR isolates and Mef(A), which confers azithromycin resistance, in one isolate.

## Discussion

With a similar clinical spectrum to Hib, NTHi has become the most common cause of invasive *H. influenzae* diseases in all age groups since the global implementation of Hib-conjugate vaccination [2]. After the isolation of the first MDR *H. influenzae* strain in 1980 [16], infections caused by these bacteria have increased rapidly, thereby triggering enhanced vigilance worldwide. Yang et al. noted a nosocomial outbreak of MDR NTHi in the respiratory care ward of a community hospital in Taiwan [13]. Yamada et al. identified an NTHi strain resistant to ampicillin, amoxicillin-clavulanate, levofloxacin, clarithromycin, and tetracycline [14]. Li et al. also identified MDR NTHi strains with β-lactam/azithromycin/trimethoprim-sulfamethoxazole as the most frequent pattern of resistance [15]. The rising incidence of NTHi in difficult-to-treat respiratory infections and the drug-resistant evolution in this microbe warrant its surveillance. The World Health Organization included *H. influenza*e on the priority pathogen list for which new antibiotics are urgently required [17]. Our study is crucial for not only yielding a better understanding of the molecular epidemiology of antibiotic resistance in NTHi but also for providing critical information on therapeutic options to confront this emerging threat.

Ampicillin remains the first-line antibiotic in the treatment of *H. influenzae* infections. However, the widespread plasmid-mediated class A serine β-lactamases, most often TEM-1 and rarely ROB-1 [18, 19], drastically reduce the efficacy of ampicillin and other β-lactam agents against *H. influenzae*. Our previous 12-year survey demonstrated that the prevalence of BLPAR and BLNAR *H. influenzae* was 30.5% and 25.5%, respectively, in Taiwan [20]. Jean et al. reported the rate of BLPAR *H. influenzae* infection was 55% in patients treated at the intensive care units of 10 major teaching hospitals in Taiwan [11]. In this study, 30.8% of the NTHi isolates were BLPAR and 15.4% were BLNAR. TEM-1 β-lactamase was detected in 75% of the BLPAR isolates. Mutations in *ftsI* may increase ampicillin resistance in BLPAR strains. Two BLPAR isolates had type I FtsI mutations (Arg517His), 3 had type II (Asn526Lys), and 3 had type III (Ser385Thr with Asn526Lys) [21–23]. This may be the reason for the higher mean MIC of ampicillin in the BLPAR isolates (58.4 μg/mL) than in the BLNAR isolates (2.3 μg/mL). BLNAR *H. influenzae* strains are also less susceptible to other β-lactam agents, particularly cephalosporins. Cefaclor or cefuroxime might be better indicators than ampicillin for BLNAR strains [24, 25]. Straker et al. identified that Ser357Asn substitution in FtsI has a profound effect on cefuroxime resistance in *H. influenzae*, while D350N, A502T, and N526K do not directly attribute to cefuroxime resistance but may play an additive role in reduced cefuroxime susceptibility when present with other substitutions [26]. No mutations in *dacA* and *dacB*, encoding PBP5 and PBP4, respectively, were associated with cefuroxime resistance [26]. All 8 cefuroxime-resistant isolates had Ser357Asn substitution in FtsI except isolate No. 23, in which Arg517His, Thr532Ala, and Tyr557His substitutions in FtsI and Met151Ile in LpoA, a PBP activator, were found. Met151Ile substitution in LpoA was also detected in 2 cefuroxime-resistant isolates. The result from logistic regression showed that Thr532Ser substitution in FtsI but not Arg517His and Ser385Thr was correlated with high-level cefuroxime resistance. However, 5 NTHi isolates harbored this mutation and type III-like mutation in FtsI simultaneously. Accordingly, Thr532Ser substitution in FtsI may greatly augment cefuroxime resistance. Three genetic variations, causing missense mutations in MrdA, MepA, and PbpG, respectively, were linked to amoxicillin-clavulanate non-susceptibility in NTHi. MrdA (PBP2) is a D,D-transpeptidase. PbpG (PBP7) is a D,D-endopeptidase that hydrolyzes diaminopimelate-alanine bonds. MepA is a penicillin-insensitive murein endopeptidase that cleaves the D-alanyl-meso-2,6- diamino-pimelyl amide bond. These proteins are essential for peptidoglycan synthesis during cell wall elongation. Further investigations are needed to understand how mutations in these proteins contribute to amoxicillin-clavulanate non-susceptibility.

Regarding macrolide resistance, *H. influenzae* may acquire efflux pumps homologous to the AcrAB efflux machinery or to major facilitator superfamily transporters in *Escherichia coli*, thereby leading to the extrusion of macrolides from cells. Although the existence of macrolide efflux genes in *H. influenzae* has been a subject of debate, several studies have confirmed their presence. Roberts et al. identified azithromycin-resistant NTHi in children with cystic fibrosis, and *mef(A)*, *erm(B)*, and *erm(F)* were detected in 74%, 31%, and 29% of isolates, respectively [27]. Moreover, *mef(A)*-bearing NTHi was reported in Japan [28]. We identified one azithromycin-resistant isolate (MIC = 8 μg/mL) with *mef(A)*. No genetic variations in the efflux pump genes were associated with azithromycin resistance in this isolate. We should be cautious regarding the expansion of macrolide-resistant NTHi because of a horizontal transfer of macrolide-resistance genes from other microbes over time. Four MDR NTHi isolates obtained conjugative plasmids with *tet(B)*, which encodes an efflux protein leading to tetracycline resistance. Tet(B) can confer resistance to both tetracycline and minocycline, but not to the new glycylcyclines [29]. Therefore, 4 *tet(B)*-positive isolates exhibited MICs of tetracycline higher than 4 μg/mL but were all susceptible to tigecycline.

Because of toxicity, quinolones are not favored as therapeutic options in the treatment of *H. influenzae* diseases, particularly in children. Resistance to quinolones in *H. influenzae* is rare in the West but severe in East Asia. In Taiwan, as indicated by earlier surveys, the levofloxacin non-susceptibility rate in *H. influenzae* is 12.5% to 14.1% [20, 30]. Herein, 57.7% of the NTHi isolates and 61.5% of MDR strains were non-susceptible to ciprofloxacin and levofloxacin. Similar to the cause of quinolone resistance in other bacterial species, resistance in NTHi mainly results from mutations in the quinolone resistance-determining region of the genes encoding DNA gyrase or topoisomerase IV [31, 32]. Studies have demonstrated that in *H. influenzae*, Ser84 and Asp88 substitutions in GyrA, and Gly82, Ser84, and Glu88 in ParC were closely related to low quinolone susceptibility [33–35]. In addition to these mutations, we identified novel significant mutations that rendered high-level levofloxacin resistance. One point mutation in the *parC*-*parE* intergenic region was detected in 73% of levofloxacin/ciprofloxacin-non-susceptible isolates but was absent in all susceptible isolates. This mutation locates in the promoter region of *parC* and how it affects gene expression is unclear. These mutations were shown to be related to high-level levofloxacin resistance in a univariate but not a multivariate regression model (data not shown), indicating that these factors were tightly correlated, which can be evidenced by the network clustering model.

Trimethoprim-sulfamethoxazole, by blocking tetrahydrofolate synthesis in bacterial cells, is a relatively inexpensive drug, with wide use worldwide. Trimethoprim resistance is caused primarily by the low affinity of mutant dihydrofolate reductase to the drug but not to natural substrates, whereas sulfamethoxazole resistance generally arises from the acquisition of either *sul1* or *sul2*, encoding different forms of dihydropteroate synthase that are not inhibited by the drug. In this study, neither *sul1*/*sul2* nor genetic variations in *folP* and *thyA* were linked to trimethoprim-sulfamethoxazole resistance in the NTHi isolates. Six substitutions in FolA identified in this study have been characterized to be associated with low-level trimethoprim-sulfamethoxazole resistance [36, 37]. These 6 substitutions individually had no relevance in high-level resistance and seemed to act in an interconnected manner to cause the higher MIC values. Our earlier report demonstrated that more than half of *H. influenzae* strains in Taiwan were resistant to trimethoprim-sulfamethoxazole, making this drug no longer efficacious in the treatment of *H. influenzae* infections [20]. Therefore, clinicians should be aware of the local trimethoprim-sulfamethoxazole resistance situation and use this drug carefully with consideration of qualified susceptibility reports.

## Conclusion

In closing, no extended-spectrum β-lactamases were detected in our NTHi isolates. Carbapenems and tigecycline are currently active against MDR NTHi strains. Studies monitoring trends in the antimicrobial resistance of *H. influenzae* should be standardized and continued. Furthermore, antibiotic stewardship at healthcare facilities should exercise standard operating procedures to manage *H. influenzae* infections, particularly those caused by MDR strains, and to avoid the acquisition of broad spectrum β-lactamases or other resistant mechanisms to last-line drugs.

## Materials and methods

### Isolation and serotyping of *H. influenzae*

The Institutional Review Board of E-Da Hospital approved this study (No. 2021009) and waived the informed consent requirement because this study used only bacterial isolates and did not have any negative impact on patients. No patients were under the age of 18 years. After the exclusion of *H. influenzae* isolates from unqualified sputum specimens, 26 NTHi isolates were collected from September 2017 to December 2019, as previously reported [38]. Eight isolates were obtained from blood cultures and 18 from sputum specimens. Suspected bacteria were re-isolated on chocolate agar (Creative Life Sciences, New Taipei city, Taiwan) and identified using matrix-assisted laser desorption ionization-time of flight mass spectrometry (VITEK MS, BioMérieux, Marcy-l’Étoile, France). Slide agglutination tests were conducted using Difco Haemophilus Influenzae Antisera (Becton, Dickinson and Company, Sparks, MD, USA) to determine the serotype of *H. influenzae*. Isolates were stored in skim milk containing glycerol at − 80 ℃ until use.

### Antimicrobial susceptibility testing

The minimum inhibitory concentrations (MICs) of 12 antimicrobial agents, namely ampicillin (penicillins), amoxicillin-clavulanate (β-lactam combination agents), cefuroxime (cephems), cefotaxime (cephems), ertapenem (carbapenems), meropenem (carbapenems), trimethoprim-sulfamethoxazole (folate pathway antagonists), azithromycin (macrolides), levofloxacin (fluoroquinolones), ciprofloxacin (fluoroquinolones), tetracycline (tetracyclines), and tigecycline (glycylcycline), were examined using ETEST strips (BioMérieux). Disk diffusion tests using a BBL Sensi-Disc (Becton, Dickinson and Company) were also performed to confirm the drug susceptibility patterns. The ATCC 49247 NTHi strain was used as the control in antimicrobial susceptibility tests. The antimicrobial susceptibility breakpoints were interpreted in accordance with the Clinical & Laboratory Standards Institute, M100, 31st Edition guidelines. However, for tigecycline, the US Food and Drug Administration breakpoints were used. MDR is defined as non-susceptibility to at least one agent in three or more antimicrobial categories [39]. High-level resistance to cefuroxime, levofloxacin, and trimethoprim-sulfamethoxazole in NTHi were defined as MIC of ≧64 μg/mL, ≧32 μg/mL, and ≧32 μg/mL, respectively.

### Genomic and plasmid DNA purification and sequencing

Each NTHi isolate was cultured on chocolate agar at 37 ℃ with 5% CO_2_ for 20 h, which was followed by harvesting. Bacterial genomic DNA and plasmid DNA were extracted using a Presto Mini gDNA Bacteria Kit (Geneaid, New Taipei City, Taiwan) and a QIAGEN Plasmid Midi Kit (QIAGEN, Germantown, MD, USA), respectively. DNA samples were concentrated using a Qubit dsDNA HS assay kit (Thermo Fisher Scientific). The DNA library was prepared using a Nextera XT DNA Library Preparation Kit (Illumina, San Diego, CA, USA) and sequenced with the MiSeq system (Illumina) using the 250-bp paired-end read protocol.

### Bioinformatic analyses

The quality of raw read data was analyzed using FastQC [40]. High-quality reads were trimmed using Trimmomatic [41] and assembled into genomes using BWA software with *H. influenzae* strain Rd KW20 as the reference (NC_000907.1). A genome subset was generated using PacBio, and Samtools was used to assess plot coverage. Variant calling and annotation were conducted using Bcftools and SnpEff, respectively. Drug-resistant genes on plasmids were assessed using BLASTx and compared with sequences available in the RefSeq database of the National Center for Biotechnology Information. Alignment of reads to plasmids was accomplished using the QIAGEN CLC Workbench software (CLC bio).

## Statistical analysis

SPSS 18.0 for Windows was used for statistical analyses. Fisher's exact tests were used to search amino acid substitutions that were associated with drug non-susceptibility. A receiver operator characteristic (ROC) curve was used for different amino acid substitutions to differentiate the MIC of antimicrobial agents. Logistic regression assays were used to identify amino acid substitutions that were associated with high-level resistance to cefuroxime and levofloxacin. Significance is set at *P* < 0.05 (2-tailed).

## Supplementary Information

**Additional file 1:** **Supplementary Table 1.** Numbers of genetic variations detected in drug resistance-associated transporter and outer membrane protein genes in NTHi isolates.

## Data Availability

Raw DNA-Seq reads of each NTHi isolate are available from the NCBI (PRJNA918521).

## Abbreviations

AUROC: The area under receiver operating characteristic curve
BLNAR: β-Lactamase-negative ampicillin-resistant
BLPAR: β-Lactamase-positive ampicillin-resistant
Hib: *Haemophilus influenzae* Serotype b
MDR: Multidrug-resistant
MIC: Minimum inhibitory concentration
NTHi: Non-typeable *Haemophilus influenzae*
PBP: Penicillin-binding protein
