# Supplementary material for: Molecular characterization of multidrug-resistant non-typeable Haemophilus influenzae with high-level resistance to cefuroxime, levofloxacin, and trimethoprim-sulfamethoxazole
Source: BMC Microbiol. 2023 Jul 5;23:178. doi: 10.1186/s12866-023-02926-6 (PMC10320927; doi:10.1186/s12866-023-02926-6)
Supplement: Supplementary file 1 — Additional file 1: Supplementary Table 1. Numbers of genetic variations detected in drug resistance-associated transporter and outer membrane protein genes in NTHi isolates. [file 12866_2023_2926_MOESM1_ESM.docx]

| Supplementary Table 1. Numbers of genetic variations detected in drug resistance-associated transporter and outer membrane protein genes in NTHi isolates | | | | | | | | |
| --- | --- | --- | --- | --- | --- | --- | --- | --- |
| Isolate | *HI_0139 (ompP2)* | *HI_0893 (acrR)* | *HI_0894 (acrA)* | *HI_0895 (acrB)* | *HI_0897 (emrB)* | *HI_0898 (emrA)* | *HI_1164 (ompP5)* | *HI_1462 (tolC)* |
| ED01 | 21 | 4 | 11 | 9 | 5 | 19 | 20 | 5 |
| ED02 | 28 | 2 | 6 | 6 | 5 | 11 | 19 | 3 |
| ED03 | 28 | 2 | 6 | 13 | 5 | 10 | 18 | 6 |
| ED04 | 29 | 1 | 6 | 6 | 5 | 11 | 19 | 3 |
| ED05 | 48 | 5 | 20 | 38 | 3 | 10 | 21 | 7 |
| ED06 | 27 | 4 | 11 | 31 | 15 | 17 | 7 | 3 |
| ED07 | 26 | 1 | 5 | 7 | 4 | 11 | 17 | 4 |
| ED08 | 31 | 1 | 5 | 7 | 4 | 11 | 20 | 4 |
| ED09 | 19 | 3 | 11 | 16 | 5 | 16 | 18 | 3 |
| ED10 | 32 | 2 | 20 | 21 | 7 | 22 | 22 | 6 |
| ED11 | 46 | 13 | 17 | 19 | 10 | 9 | 31 | 15 |
| ED12 | 56 | 13 | 23 | 23 | 13 | 24 | 35 | 10 |
| ED13 | 58 | 11 | 19 | 26 | 19 | 31 | 23 | 7 |
| ED14 | 53 | 12 | 23 | 21 | 14 | 18 | 33 | 5 |
| ED15 | 39 | 3 | 13 | 18 | 6 | 18 | 23 | 5 |
| ED16 | 59 | 6 | 32 | 41 | 12 | 16 | 23 | 9 |
| ED17 | 61 | 11 | 32 | 36 | 10 | 19 | 27 | 6 |
| ED18 | 30 | 4 | 24 | 39 | 11 | 19 | 31 | 8 |
| ED19 | 37 | 7 | 18 | 8 | 5 | 20 | 17 | 4 |
| ED20 | 50 | 7 | 25 | 24 | 11 | 21 | 11 | 6 |
| ED21 | 72 | 6 | 39 | 27 | 7 | 17 | 30 | 11 |
| ED22 | 54 | 3 | 11 | 23 | 10 | 23 | 35 | 7 |
| ED23 | 55 | 6 | 34 | 30 | 10 | 19 | 43 | 10 |
| ED24 | 57 | 6 | 23 | 30 | 12 | 23 | 28 | 10 |
| ED25 | 68 | 9 | 40 | 34 | 9 | 20 | 20 | 13 |
| ED26 | 29 | 5 | 28 | 38 | 10 | 24 | 24 | 6 |
| *Haemophilus influenzae* Rd KW20 was used as the reference strain. NTHi, non-typeable *H. influenzae*. | | | | | | | | |
